# Early-life nutritional status as a determinant of bone density and hip joint morphology in children

**DOI:** 10.3389/fnut.2026.1797867

**Published:** 2026-06-01

**Authors:** Hongliang Liu, Weizhang Zhang, Guangxi Chen, Yang Lv, Qiang Li, Shuchai Xu, Da Guo, Weiming Yang, Yan Liu

**Affiliations:** 1 Orthopaedics, The Second Affiliated Hospital of Guangzhou University of Traditional Chinese Medicine, Guangzhou, China; 2 Department of Emergency, The Second Affiliated Hospital of Guangzhou University of Traditional Chinese Medicine, Guangzhou, China

**Keywords:** bone mineral density, childhood skeletal health, early-life nutrition, hip joint morphology, pediatric nutrition, retrospective study

## Abstract

**Background:**

Early childhood is an important period of skeletal development during which nutritional exposures can affect mineral accretion and joint morphology. Nonetheless, few studies have compared bone mineral density (BMD) and hip joint morphology in children.

**Objectives:**

The aim of the study is to investigate the relationship between the nutritional status of children at an early age and the BMD and morphology of hip joints in children.

**Methods:**

It was a retrospective cohort study of 550 children aged 614 years who had the data of dual-energy X-ray absorptiometry and hip radiography (January 2021–December 2025). The nutritional status of infants in early life was defined as adequate or suboptimal based on the infant’s feeding practices and developmental indicators. Regression analyses were performed using multivariate models, adjusting for age, sex, birth weight, BMI, gestational age, physical activity, and vitamin D supplementation.

**Results:**

The children who were fed poorly in their early life had poorer total-body and site-specific *Z*-scores of BMD and a more prevalent incidence of altered hip morphology. After adjustment, nutritional optimization remained strongly associated with low BMD and increased risk of low bone density and hip deformities. There was an inverse-proportional relationship, graded, whereby low nutritional scores were associated with low skeletal performance.

**Conclusion:**

Nutritional status in early-life was found to be significantly connected with the bone mineral density and morphology of hip joints in childhood. The results indicate that early nutritional exposures may play an important role in children’s skeletal health, but it is impossible to draw causal conclusions because the study is retrospective.

## 1 Introduction

Childhood is a period of skeletal growth and development during which bones are deposited, and joints form very quickly. At this age, nutrition should be appropriate to achieve optimal bone mineral density (BMD), which is one of the most important factors in bone strength and resistance to fractures throughout life. Even in early childhood, pediatric cohort studies have demonstrated that diet intake, body composition, and physical activity significantly impact BMD outcomes. The findings emphasize how sensitive the developing skeleton is to nutritional and lifestyle exposures during early life; therefore, preventive measures should be implemented early to maximize bone health. There is also emerging evidence that maternal nutritional and metabolic conditions during pregnancy support fetal skeletal development and that these conditions are associated with long-term bone health (1).

Micronutrients (calcium and vitamin D) and macronutrients (carbohydrates, fats, and proteins) have direct effects on the formation and deposition of bone tissue. As longitudinal data show, the factors affecting BMD can be observed even in six-month-old children and continue to influence it into early childhood, suggesting that the effect of nutrition on bone health may be traced back to extremely young ages. Along with individual nutrient intake, general eating habits have also been associated with bone mass in children. Healthy dietary practices, including a high intake of nutrient-rich foods such as dairy, fruits, vegetables, and whole grains, are associated with better bone health outcomes among school-aged and pre-adolescent boys and girls. On the other hand, the quality of the diet might have a detrimental effect on the bone mass accrual at sensitive developmental stages (2).

Multifactorial skeletal development is further supported by population-based pediatric research, which has shown that bone health is related to the interaction among diet quality, lifestyle, and anthropometric factors (3, 4). Maternal nutrition and perinatal factors are also known to affect skeletal development in children at early stages and bone density. The quality of nutrient intake in the course of pregnancy can directly influence the development of bones in the fetus, and poor maternal diet consumption has been associated with low BMD in children. Observational and interventional studies have shown that prenatal nutrition, gestational growth, and early postnatal nutrition all contribute to bone health in early life, supporting a life-course perspective in which early nutritional exposures have long-term effects on skeletal integrity (5).

Other important effects of nutrition in early life beyond BMD include skeletal morphology, especially growth of the hip joints, but this has received little research attention. Normal locomotion, load-bearing, and long-term musculoskeletal functions of the hip require proper morphology of the hip joint. Nominal data on infant hip development indicate that early hip growth affects hip structure and geometry, implying that infant nutritional status may also contribute to variation in joint morphology (6). Recent narrative syntheses and systematic reviews indicate the effects of nutrition, physical activity, and interventions during childhood on children’s bone health. It has been proven that skeletal development can be maximized by modifiable factors in early childhood. Although research linking nutrition to bone density has increased, few studies have examined both BMD and hip joint morphology in children, warranting further research (7, 8).

Although nutritional status in early life is a major factor associated with pediatric bone mineral density, existing research focuses predominantly on bone density outcomes and rarely considers hip joint morphology as a structural measure of skeletal growth. There is still a lack of evidence regarding how early childhood nutritional exposure relates to bone density and hip joint geometry within the same population of children. Consequently, the proposed study will assess early-life nutritional status as an associated factor of bone density and hip joint morphology in children, contributing to a more comprehensive picture of skeletal health in childhood and informing early-life prevention measures. To determine nutritional status in early life by determining dietary and nutritional indicators, and to assess bone mineral density in children and determine its relationship with early-life nutritional status, and measure parameters of the morphology of hip joints, and find out how these parameters are related to early-life nutritional status.

## 2 Methodology

### 2.1 Study design

The study was a retrospective observational cohort study that aimed to establish associations between early-life nutritional status and bone mineral density and hip joint morphology in children. The retrospective design allowed assessment of recorded infant nutritional exposures (0–24 months) and their relationship with childhood skeletal outcomes, consistent with epidemiological approaches in pediatric bone health research.

### 2.2 Study setting and duration

The research was conducted at a tertiary-level teaching hospital, using archived records from medicine, nutrition, and radiology for pediatric patients. Retrospective data collection was conducted between 2021 and 2025 and included records of children with documented early-life nutritional data (0–24 months) and subsequent measurements of bone mineral density and hip joint morphology during childhood. This was a period during which they achieved sufficient nutritional exposure within the first 2 years of life, and their relationship with skeletal outcomes was measured in childhood.

### 2.3 Study population and sample size

The study involved 550 children. Children were aged between 6 and 14 years at the time of bone mineral density and radiographic assessment. Age was determined from the imaging and clinical evaluation records. Both male and female participants were included. Hospital records were used to sample participants based on consecutive sampling. The sample size was sufficiently large to yield statistically significant results on the associations between early nutritional status and skeletal outcomes and to control for the effects of crucial confounders.

### 2.4 Inclusion and exclusion criteria

The inclusion of children was based on the availability of a complete record of nutritional status during the first 2 years of life, at least 1 bone mineral density measurement, and x-ray evidence of the hip joint from childhood. Term-born children only were included to reduce confounding due to the skeletal effects of prematurity. The children who were excluded from the analysis were those with chronic systemic diseases that affect bone metabolism, genetic skeletal disorders, who had taken long-term medications that are known to affect bone health, and those with incomplete medical records.

### 2.5 Assessment of early life nutritional status

Nutritional status in early life (0–24 months) was determined using a composite method that combined reported infant feeding habits and standardized growth measures. The variables to be fed on were the time that the infant is breastfed, the time that the infant is fed the complementary food, the variety of the diet and the consumption of foods rich in calcium and supplementation of vitamin D. The weight-for-age, length-for-age, and weight-for-length *Z*-scores were assessed to evaluate the growth indicators founded on the WHO Child Growth Standards that are popular in the pediatric nutritional evaluation and growth measurement (5, 9).

To enhance reproducibility, a highly structured scoring system was developed in which essential nutritional measurements were assigned binary scores (adequate = 1, suboptimal = 0). The range of the cumulative nutritional score was 0–6. Children with a score of 4 or above were considered to have relatively adequate nutrition, whereas those with a score below 4 were considered to have relatively suboptimal nutrition, as in earlier pediatric nutrition and growth surveys that have assessed composite dietary exposures (10).

Although nutritional exposure is continuous, it was categorized to enable clinical interpretability. Continuous indicators (e.g., number of favorable nutritional exposures) were also subjected to sensitivity analyses to investigate the possible dose–response relationships, as was exemplified in nutritional epidemiology literature (2, 11). Investigators who were not involved in radiological outcome assessment classified nutritional exposure using archived records, thereby minimizing the possibility of classification bias. This mixed-methods approach has been adopted in some previous observational studies of children in which quantitative data on dietary components are unavailable, and a pragmatic yet standardized categorization of early nutritional exposures is necessary (4, 10).

### 2.6 Bone mineral density assessment

The bone mineral density data were obtained from available dual-energy x-ray absorptiometry records obtained in childhood. They are the values of areal bone mineral density and *Z*-scores, age- and sex-adjusted. The bone density results were compared with established pediatric reference ranges, and low bone density was identified using accepted *Z*-score thresholds. All measures were obtained through routine clinical evaluation procedures as part of pediatric care.

### 2.7 Assessment of hip joint morphology

Hip joint morphology was evaluated using existing pelvic radiographs and radiographic reports. Standardized radiographic parameters reflecting acetabular development and femoral head alignment, including acetabular index, femoral head coverage, and center-edge angle, were assessed. Based on age-adjusted reference ranges, hip morphology was classified as normal, mildly altered, or moderately altered. All developmental abnormalities were documented, and image interpretation and classification were performed by experienced radiologists, who were blinded to participants’ nutritional status to minimize assessment bias. Radiologists were blinded to the status of early-life nutritional exposure.

### 2.8 Covariates and confounding factors

Medical records were used to obtain data on potential confounding variables: age, sex, birth weight, body mass index at the time of assessment, and reported physical activity or mobility status. Data on vitamin D supplementation and intermediate-diagnosed hip conditions were also collected where available. The analytical models considered these variables in case they could affect the bone and hip outcomes. Quantitative data on nutrient intake (e.g., amounts of calcium, protein, and vitamin D consumed per day) were not always available; therefore, proxy measures were used (e.g., dietary patterns and supplementation history).

### 2.9 Statistical analysis

The standard statistical software was used to perform the statistical analysis (SPSS 26.0). Participant characteristics and outcome measures were summarized using descriptive statistics. Comparisons of early-life nutritional status groups based on data distribution were used to test differences in bone mineral density and hip morphology. Multivariate regression tests were performed to assess whether there is a significant independent association between early-life nutritional status and bone mineral density, and logistic regression models were employed to examine the association between nutritional status and changes in hip structure. All analyses were adjusted for all covariates, and a two-sided *p*-value < 0.05 was considered statistically significant. Besides binary classification, the cumulative nutritional score was used as a continuous variable to assess possible dose–response relationships through the exploratory analyses. In secondary analyses, Bonferroni-adjusted significance levels were used to account for the multiple comparisons. Moreover, more attention was paid to effect estimates and their corresponding confidence intervals rather than the single use of *p*-values, which is also consistent with current recommendations for statistical reporting (12, 13).

### 2.10 Ethical considerations

This research was approved by the Ethics Committee of the Second Affiliated Hospital of Guangzhou University of Traditional Chinese Medicine, with the approval number ZF2023-007. The institutional ethics review committee gave ethical approval to the study. Since the research consisted of the retrospective review of available medical records, informed consent was not necessary. To maintain confidentiality and adhere to ethical principles, all data were anonymized before analysis.

## 3 Results

### 3.1 Participants characteristics

The resulting analysis included 550 children with complete nutritional, densitometric, and radiological information. Table 1 showed that the respondents were classified based on their early-life nutritional condition as adequate nutrition (*n* = 332) and suboptimal nutrition (*n* = 218). The average age in skeletal examination was similar across groups. There were no significant differences in sex distribution. Those children with less-than-optimal nutrition in the early stages of life demonstrated poorer developmental outcomes in infancy and a small but statistically significant shift toward lower BMI categories at the time of assessment.

**Table 1:** Baseline characteristics of the study population by early-life nutritional status (*n* = 550).

Variable	Adequate nutrition (*n* = 332)	Suboptimal nutrition (*n* = 218)	*p*-value
Age at assessment (years), mean ± SD	10.2 ± 2.4	10.0 ± 2.6	0.38
Male sex, *n* (%)	168 (50.6)	114 (52.3)	0.69
Female sex, *n* (%)	164 (49.4)	104 (47.7)	0.69
Gestational age < 37 weeks, *n* (%)	29 (8.7)	41 (18.8)	<0.001
Birth weight < 2.5 kg, *n* (%)	42 (12.7)	56 (25.7)	<0.001
Small for gestational age, *n* (%)	31 (9.3)	47 (21.6)	<0.001
Exclusive breastfeeding ≥ 4 months, *n* (%)	214 (64.5)	92 (42.2)	<0.001
Predominant formula feeding in infancy, *n* (%)	61 (18.4)	83 (38.1)	<0.001
Delayed complementary feeding (>6 months), *n* (%)	38 (11.4)	71 (32.6)	<0.001
Low dietary diversity in infancy, *n* (%)	49 (14.8)	79 (36.2)	<0.001
Vitamin D supplementation in infancy, *n* (%)	201 (60.5)	89 (40.8)	<0.001
Calcium-rich food intake reported in infancy, *n* (%)	186 (56.0)	74 (33.9)	<0.001
BMI below age-specific median at assessment, *n* (%)	86 (25.9)	83 (38.1)	0.003
Low physical activity level, *n* (%)	72 (21.7)	69 (31.7)	0.01

Children with inadequate early-life nutrition were associated with lower birth weight, reduced breastfeeding exposure, delayed complementary feeding, and lack of vitamin D supplementation, which may reflect suboptimal nutritional conditions during infancy, although other contributing factors cannot be excluded.

### 3.2 Bone mineral density outcomes

Bone mineral density results varied widely depending on nutritional status in the early stages of life. Children with sufficient nutrition had higher mean BMD *Z*-scores at axial and appendicular skeletal locations, and the frequency of low bone density was significantly higher in children with suboptimal nutrition. Such clinical differences remained significant even after adjusting for age, sex, BMI, and birth weight (Table 2).

**Table 2:** Bone mineral density parameters by early-life nutritional status.

BMD parameter	Adequate nutrition (*n* = 332)	Suboptimal nutrition (*n* = 218)	*p*-value
Total body BMD (g/cm^2^), mean ± SD	0.82 ± 0.09	0.76 ± 0.11	<0.001
Total body BMD *Z*-score, mean ± SD	−0.18 ± 0.72	−0.61 ± 0.81	<0.001
Lumbar spine BMD (g/cm^2^), mean ± SD	0.71 ± 0.08	0.66 ± 0.10	<0.001
Lumbar spine BMD *Z*-score, mean ± SD	−0.12 ± 0.68	−0.55 ± 0.77	<0.001
Femoral neck BMD (g/cm^2^), mean ± SD	0.74 ± 0.07	0.69 ± 0.09	<0.001
Femoral neck BMD *Z*-score, mean ± SD	−0.21 ± 0.70	−0.66 ± 0.83	<0.001
Whole-body lean mass–adjusted BMD *Z*-score, mean ± SD	−0.09 ± 0.66	−0.48 ± 0.74	<0.001
Normal BMD (*Z* ≥ −1), *n* (%)	284 (85.5%)	142 (65.1%)	<0.001
Low BMD (*Z* < −1), *n* (%)	48 (14.5%)	76 (34.9%)	<0.001
Very low BMD (*Z* < −2), *n* (%)	11 (3.3%)	29 (13.3%)	<0.001

Children with suboptimal infant nutrition had much smaller BMI reductions and *Z*-scores at both axial and appendicular sites. The prevalence of low and very low BMD groups in this group is an important indicator of the long-term skeletal effects of early nutritional deficiency.

### 3.3 Hip joint morphology

Radiological assessment of the subjects showed that hip joint structural parameters differed across groups according to early-life nutrition. Those children who were poorly fed during infancy showed more frequent acetabular and femoral anomalies, as well as angular deviations indicating altered hip biomechanics. The differences were also the same in the unilateral and bilateral tests (Table 3).

**Table 3:** Hip joint morphology parameters by early-life nutritional status (*n* = 550).

Hip morphology variable	Adequate nutrition (*n* = 332)	Suboptimal nutrition (*n* = 218)	*p*-value
Normal hip morphology, *n* (%)	297 (89.5%)	161 (73.9%)	<0.001
Any hip abnormality, *n* (%)	35 (10.5%)	57 (26.1%)	<0.001
Laterality of abnormality			
Unilateral	21 (6.3%)	33 (15.1%)	<0.001
Bilateral	14 (4.2%)	24 (11.0%)	0.002
Acetabular index above age norm, *n* (%)	26 (7.8%)	41 (18.8%)	<0.001
Shallow acetabulum, *n* (%)	19 (5.7%)	34 (15.6%)	<0.001
Reduced femoral head coverage, *n* (%)	18 (5.4%)	37 (17.0%)	<0.001
Increased neck–shaft angle, *n* (%)	12 (3.6%)	29 (13.3%)	<0.001
Delayed femoral head ossification, *n* (%)	9 (2.7%)	23 (10.6%)	<0.001
Radiological severity classification			
Mild alteration	24 (7.2%)	39 (17.9%)	<0.001
Moderate alteration	9 (2.7%)	15 (6.9%)	0.01
Marked alteration	2 (0.6%)	3 (1.4%)	0.31

Children who were poorly fed during their early years exhibited very high rates of acetabular shallowness, covered femur heads, and angular proximal femoral deviations. Bilateral intervention and intermediate structural changes were more prevalent in this sample, which may indicate that nutritional ineffectiveness in early development can affect hip development and symmetry.

### 3.4 Relationship between bone density and hip morphology

The hip morphology of children showed a continuous decrease in bone mineral density across various skeletal locations compared with those with normal hip structure. This correlation was graded, and the lower the BMD values, the more severe the morphological change in the hip, as shown in Table 4. The correlation was maintained within the groups of early-life nutritional factors, but was strongest among children who experienced suboptimal nutrition during infancy, suggesting a possible interaction between joint maturation and systemic bone mineralization.

**Table 4:** Bone mineral density parameters according to hip joint morphology.

Hip morphology category	Total BMD *Z*-score (mean ± SD)	Lumbar spine BMD *Z*-score (mean ± SD)	Femoral neck BMD *Z*-score (mean ± SD)	Low BMD (*Z* < −1), *n* (%)	Very low BMD (*Z* < −2), *n* (%)	*p*-value
Normal hip	−0.26 ± 0.74	−0.21 ± 0.70	−0.29 ± 0.76	92 (19.8)	21 (4.5)	–
Mild alteration	−0.58 ± 0.79	−0.52 ± 0.75	−0.61 ± 0.82	38 (38.0)	12 (12.0)	<0.001
Moderate alteration	−0.83 ± 0.88	−0.79 ± 0.84	−0.91 ± 0.90	21 (55.3)	9 (23.7)	<0.001

*p*-value represents the comparison across morphology categories.

Children with mild and moderate hip morphological changes had very low total body, lumbar spine, and femoral neck BMD *Z*-scores compared with children with normal hips. There was an increase in the prevalence of low and very low bone density in parallel with the degree of morphology, which favors a dose–response association between hip joint growth and skeletal mineralization.

### 3.5 Multivariable association between early-life nutrition and skeletal outcomes

Table 5 presents multivariate regression models used to determine the significant association between early-life nutritional status and skeletal outcomes, controlling for age, sex, body mass index, gestational age, birth weight, physical activity level, and vitamin D supplementation. Nutritional status in early life was a prominent associated factor for bone mineral and hip joint morphology across all adjusted models.

**Table 5:** Multivariable regression analysis of early-life nutritional status and skeletal outcomes.

Outcome variable	Adjusted effect estimate	95% confidence interval	*p*-value
Total body BMD *Z*-score (*β* coefficient)	−0.34	−0.48 to −0.20	<0.001
Lumbar spine BMD *Z*-score (*β* coefficient)	−0.31	−0.44 to −0.18	<0.001
Femoral neck BMD *Z*-score (*β* coefficient)	−0.37	−0.52 to −0.22	<0.001
Low BMD (*Z* < −1), odds ratio	2.1	1.5–3.0	<0.001
Very low BMD (*Z* < −2), odds ratio	2.8	1.6–4.9	<0.001
Any hip morphological abnormality, odds ratio	2.4	1.6–3.5	<0.001
Moderate-to-marked hip alteration, odds ratio	2.7	1.4–5.2	0.003

Suboptimal nutritional intake in the first child years was also an independent factor associated with reduced bone mineral density at both axial and appendicular skeletal locations and an increased risk of mild and more severe hip joint abnormalities. These results suggest that early nutritional exposure has long-term effects on bone mass and joint morphology that are not related to growth, body composition, or other lifestyle-related factors.

### 3.6 Combined skeletal outcomes

The joint presence of low bone mineral density and changes in hip morphology was measured to identify the children with the poorest skeletal phenotype. The children who had received suboptimal nutrition during infancy were overrepresented in the combined adverse outcome categories that were a sign of a cumulative skeletal burden (Table 6).

**Table 6:** Combined bone density and hip morphology outcomes by early-life nutritional status.

Skeletal profile	Adequate nutrition *n* (%)	Suboptimal nutrition *n* (%)	*p*-value
Normal BMD + normal hip morphology	268 (80.7%)	128 (58.7%)	<0.001
Normal BMD + altered hip morphology	29 (8.7%)	37 (17.0%)	<0.001
Low BMD + normal hip morphology	19 (5.7%)	21 (9.6%)	0.04
Low BMD + altered hip morphology	16 (4.8%)	32 (14.7%)	<0.001
Very low BMD + altered hip morphology	4 (1.2%)	14 (6.4%)	<0.001

Children who had experienced suboptimal nutrition in childhood were more likely to report combined skeletal deficits, including low or very low bone mineral density and altered hip morphology. This trend indicates a cumulative negative impact of early nutritional compromise on bone strength and joint structure.

## 4 Discussion

The current article showed that children who endured poor nutritional conditions in their early life experienced far worse perinatal and infancy-related outcomes, which included higher rates of prematurity, low birth weight, small-for-gestational-age, lower exclusive breastfeeding, delayed complementary feeding, and worse micronutrient exposure. The findings affirm the long-standing correlation in early nutrition theory, in which the adverse effects of poor nutrition during critical developmental stages are detrimental to the development of the soma and skeletal health (14). This trend of decreased BMI and sedentary lifestyles in children exposed to poor nutrition during the early years also indicates long-term metabolic and growth-related consequences that persist into the pediatric years, further underscoring the significance of early nutrition as a risk factor for musculoskeletal development (9).

Children whose nutrition during the first part of their lives was suboptimal had lower bone mineral density scores at the total body, lumbar spine, and femoral neck sites, and the likelihood of low or very low *Z*-scores was much higher (15). These variations persisted even after accounting for important confounders, indicating that early nutrition is directly correlated with bone mass acquisition. These findings are consistent with research indicating that early dietary intake, calcium intake, vitamin D supplement intake, and infant and early childhood protein exposure are critical determinants of bone mineral accrual during growth (16). Poor nutrition during infancy is likely to hamper the achievement of optimal bone mass, placing future individuals at risk of osteopenia and osteoporosis (17).

Radiological analysis revealed that children who were poorly fed in early childhood had a very high rate of hip joint deformities, including shallow acetabula, high acetabular indices, low femoral head coverage, delayed ossification, and changes in neck-shaft position. These findings suggest that a dietary deficiency during early skeletal engineering may alter normal morphogenesis and biomechanical orientation of the hip joint (18). The increasing levels of bilateral interaction and the moderate structural transformation that have occurred indicate a systemic rather than localized impairment of development (11). Impaired nutrition has been shown to influence cartilage maturation, the ossification period, and joint geometry, thereby predisposing to abnormal hip development (19).

They found that bone mineral density had a graded inverse relationship with the extent of structural change in hip joint morphology, such that lower bone mineral density values indicated greater abnormality of structural change (20). This dose–response association confirms that the biological correlation between the system and bone mineralization and joint development is evident (21). Although in the present study, the nutritional exposure was classified to improve the interpretation of clinical data, the relationship is likely continuous. Additional studies involving detailed quantitative nutritional assessments are better suited to describing dose–response and provide more precise approximations of the nutritional effect on skeletal development (22). Reduced bone density can compromise the normal development of the acetabulum and femur (23).

Reduction in bone density may compromise the structural integrity of normal acetabular and femoral development, and disruption of hip biomechanics may further exacerbate local bone deficits (24). The findings are consistent with the available literature, suggesting that children with hip dysplasia or deviated acetabular geometry tend to have low bone mass, thereby linking skeletal density to joint morphology (25). Although the inverse relationship between hip joint morphology and bone mineral density indicates a structural and biomechanical correlation, the current study also demonstrates that childhood nutrition is crucial in determining this correlation among children (26).

The ongoing study revealed a significant decrease in bone mineral density among children who were poorly nourished in early childhood. These findings are in line with previous longitudinal studies that found that the quality of early diets and the adequacy of micronutrient intake influence bone mineral accrual during growth (27). Adequate nutrition during infancy likely enables osteoblasts to remain active, mineralize, and achieve optimal bone mass, whereas nutrient deficiency can inhibit skeletal mineralization (28). Multivariate regression revealed that nutritional status at an early age is not influenced by growth parameters, physical activity, gestational parameters, or vitamin D supplementation, but is an independent predictor of low bone mineral density and an increased risk of hip joint abnormalities (29).

These results show that early nutritional exposure has skeletal effects beyond postnatal lifestyle and body composition (30). The intensity of correlations between BMD deficits and alterations in hip morphology underscores the primary importance of adequate nutrition during infancy, with consideration of the optimal skeletal developmental pathway and joint integrity (31). The children who suffered poor nutrition in their early-life period were overrepresented in the individuals who had combined adverse skeletal phenotypes, i.e., low or extremely low bone mineral density and abnormal hip morphology (10). This accumulating skeletal load indicates the interdependence of bone strength and joint structure and implies a higher risk of fracture, gait changes, and degenerative joint disease in the long run (32).

In addition to decreased bone density, we observed increased hip morphological changes in children with nutritional deficiencies, a finding supported by the pediatric orthopedic literature, which shows that poor development during early growth may disrupt acetabular development and joint positioning (33). These results, combined with existing evidence that early nutritional exposures influence systemic bone mass and joint morphology, and that musculoskeletal outcomes warrant a holistic approach, indicate that musculoskeletal outcomes are interconnected and cannot be investigated in isolation (34).

### 4.1 Strengths of the study

This research has several strengths. First, it assessed skeletal health using a dual-outcome (bone mineral density and hip joint morphology) method, which is more comprehensive for assessing musculoskeletal development than studies that examine bone density only. Second, the sample size was comparatively very large, which increased the statistical power and strength of the results. Third, the use of real-world clinical data enhances the relevance of the findings to routine pediatric practice. Also, major confounders were accounted for in multivariate analyses, enabling further validation of the observed relationships between early-life nutritional status and skeletal outcomes.

### 4.2 Limitations of the study

The study has some limitations despite its strengths. The retrospective design hinders causal inference and relies on the completeness and accuracy of medical records. Consistent quantitative data on dietary intake were not always available, which may have led to misclassification of early-life nutritional status. Moreover, the research was conducted at a single center, and the findings may not be generalizable to the general population. It may not be possible to eliminate residual confounding due to unmeasured variables that include physical activity levels, genetic factors, or socioeconomic status. This was due to the lack of specific quantitative dietary intake data (e.g., calcium, vitamin D, and protein), which made assessing nutrient-specific effects difficult and possibly led to exposure misclassification. Multiple-comparison adjustment had also been carried out to minimize the risk of Type I error.

### 4.3 Future directions

Prospective longitudinal designs should be adopted in future studies to elucidate better temporal and cause-and-effect relationships between early-life nutrition and skeletal development. Generalizability would be enhanced through multicenter research with varied groups of people. The use of more comprehensive dietary measurements, biochemical nutritional parameters, and genetic information might also help to explain underlying mechanisms. Interventional studies of the potential to improve bone density and hip morphology through early nutritional optimization are also justified.

## 5 Conclusion

In summary, as this retrospective study indicates, early-life nutritional status is strongly linked to bone mineral density and hip joint morphology in children. Poor nutrition in infancy was also associated with reduced BMD and an increased rate of hip morphological changes. Although these results may indicate a correlation between early nutritional intake and skeletal growth, they do not support a causal interpretation. These associations and underlying mechanisms require further research to be explained.

## Data Availability

The raw data supporting the conclusions of this article will be made available by the authors, without undue reservation.
